# Bis­(η^2^-ethyl­ene)(η^5^-inden­yl)iridium(I)

**DOI:** 10.1107/S1600536813025300

**Published:** 2013-09-21

**Authors:** Joseph S. Merola

**Affiliations:** a Department of Chemistry 0212, Virginia Tech, Blacksburg, VA 24061, USA

## Abstract

The asymmetric unit of the title compound, [Ir(C_9_H_7_)(C_2_H_4_)_2_], consists of two independent mol­ecules. The bonding between iridium and the five-membered ring of the indenyl ligand shows the usual asymmetry associated with the typical ring slippage responsible for the enhanced activity of indenyl metal compounds when compared with the analogous cyclo­penta­dienyl metal compound. There are three short Ir—C bonds of 2.210 (3), 2.190 (4) and 2.220 (3) Å and two long Ir—C bonds to the C atoms that are part of the fused six-membered ring of 2.349 (4) and 2.366 (3) Å for one of the independent mol­ecules [2.208 (4), 2.222 (3), 2.197 (4) Å for the short distances and 2.371 (3) and 2.358 (3) Å for the long distances in the second mol­ecule]. This results in both indenyl ligands being slightly kinked, with dihedral angles of 6.8 (4)° and 6.5 (4)°.

## Related literature

For the structures of the analogous rhodium(I) complex determined from single crystal X-ray data, see: CCDC:576585 (Marder *et al.*, 1987 ▶); CCDC:567925 (Mlekuz *et al.*, 1986 ▶). For a variable temperature NMR study of the title compound, see: Szajek *et al.* (1991 ▶). The structure of an η^3^-indenyliridium complex can be found in CCDC:563532 (Merola *et al.*, 1986 ▶). For seminal discussions on the "indenyl effect" see: Hart-Davis *et al.* (1970 ▶); Rerek *et al.* (1983 ▶). The synthesis of [Ir(C_2_H_2_)_2_Cl]_2_ can be found in Herde *et al.* (1974 ▶).
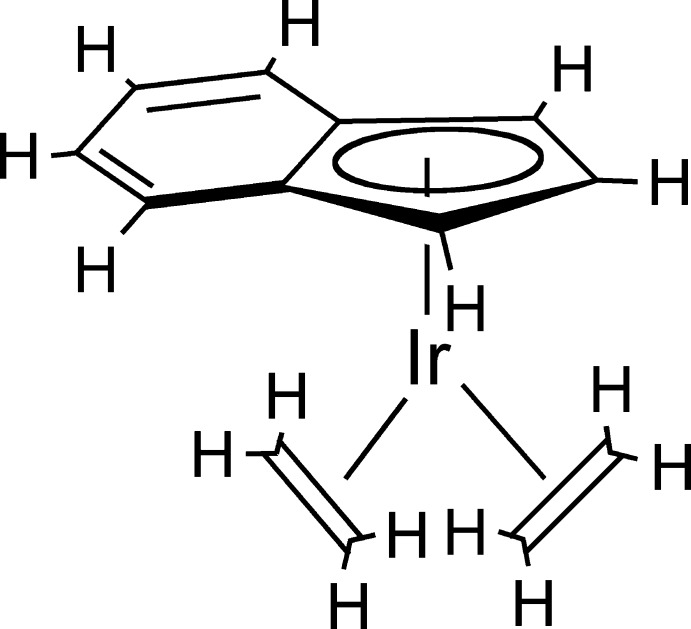


## Experimental

### 

#### Crystal data

[Ir(C_9_H_7_)(C_2_H_4_)_2_]
*M* *_r_* = 363.45
Monoclinic, 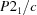

*a* = 7.73182 (11) Å
*b* = 10.77708 (13) Å
*c* = 25.6818 (5) Å
β = 98.4034 (15)°
*V* = 2117.00 (5) Å^3^
*Z* = 8
Mo *K*α radiation
μ = 12.57 mm^−1^
*T* = 100 K
0.45 × 0.33 × 0.22 mm

#### Data collection

Agilent Xcalibur, Sapphire2 diffractometer
Absorption correction: gaussian (*CrysAlis PRO*; Agilent, 2013 ▶) *T*_min_ = 0.020, *T*_max_ = 0.142
55683 measured reflections
6917 independent reflections
6733 reflections with *I* > 2σ(*I*)
*R*_int_ = 0.027

#### Refinement

*R*[*F* ^2^ > 2σ(*F* ^2^)] = 0.024
*wR*(*F* ^2^) = 0.055
*S* = 1.46
6917 reflections
253 parameters
H-atom parameters constrained
Δρ_max_ = 1.69 e Å^−3^
Δρ_min_ = −2.04 e Å^−3^

### 

Data collection: *CrysAlis PRO* (Agilent, 2013 ▶); cell refinement: *CrysAlis PRO*; data reduction: *CrysAlis PRO*; program(s) used to solve structure: *SHELXS97* (Sheldrick, 2008 ▶); program(s) used to refine structure: *SHELXL2013* (Sheldrick, 2008 ▶); molecular graphics: *OLEX2* (Dolomanov *et al.*, 2009 ▶); software used to prepare material for publication: *OLEX2*.

## Supplementary Material

Crystal structure: contains datablock(s) I. DOI: 10.1107/S1600536813025300/pk2496sup1.cif

Structure factors: contains datablock(s) I. DOI: 10.1107/S1600536813025300/pk2496Isup2.hkl

Supplementary material file. DOI: 10.1107/S1600536813025300/pk2496Isup3.mol

Additional supplementary materials:  crystallographic information; 3D view; checkCIF report

## Figures and Tables

**Fig. 1.**
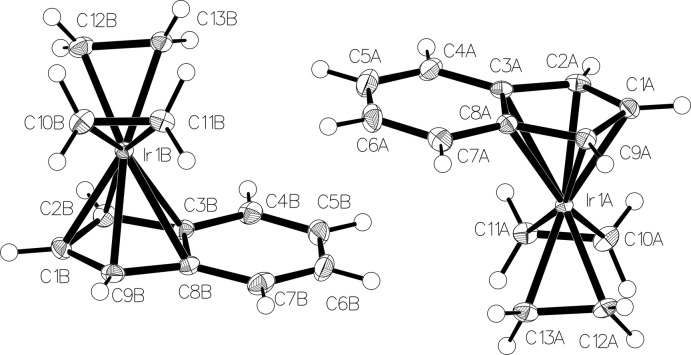
Thermal ellipsoid plot of the two indepenent molecules of the title compound. Ellipsoids are shown at 50% probability.

**Fig. 2.**
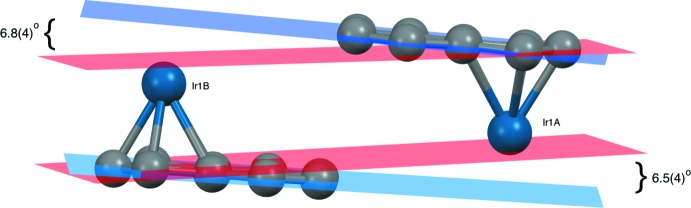
Ball and stick drawing of title compound showing the fold angle of the indenyl rings for both independent molecules. Ethylene ligands and hydrogen atoms omitted for clarity.

## supplementary crystallographic information

### 1. Comment

The indenyl ligand has been shown to be very flexible in terms of its coordination to metals. An increased reactivity that is displayed by indenyl metal complexes compared with cyclopentadienyl complexes has been dubbed the "indenyl effect". The effect was first described by Mawby's group (Hart-Davis *et al.*, 1970) and was further quantified by Basolo's group (Rerek *et al.*, 1983) We have previously reported on the synthesis and structure of η^3^-indenyliridium complexes formed by reaction of an η^5^-indenyliridiumbis(olefin) complex and small phosphine ligands such as PMe_3_ or PhPMe_2_ (Merola *et al.*, 1986). The smallest olefin complex of indenyl iridium, (η^5^-Indenyl)bis(η^2^-ethylene)iridium(I), 1, is the subject of this report. The thermal ellipsoid plot for both independent molecules of 1 is shown in figure 1. The most interesting aspects of the bonding are highlighted in table 1 showing the three short and two long bond distances of the "slipped" indenyl rings.

Figure 2 shows the "fold" of the indenyl ligand which imparts non-planarity of the 6-membered ring from the 5-membered ring. The angle between the planes defined by C1, C2 and C9 and that defined by C3, C8, C7, C4, C5 and C6 is 6.5 (4)° and 6.8 (4)° for the "A" and "B" molecules.

### 2. Experimental

[Ir(C_2_H_2_)_2_Cl]_2_ was synthesized by the reaction between [Ir(C_8_H_14_)_2_IrCl]_2_ and ethylene (Herde *et al.*, 1974). The title compound was prepared by the reaction between lithium indenide and [Ir(C_2_H_2_)_2_Cl]_2_ in anhydrous THF. Crystals of the title compound were grown by the slow evaporation of a pentane solution. The title compound has also been reported previously prepared by this same method (Szajek *et al.*, 1991).

### 3. Refinement

Crystal data, data collection and structure refinement details are summarized in Table 1. Hydrogen atoms were found in difference maps and refined using a riding model with C-H distances of 0.93 Å (C_indenyl_) and 0.97 Å (C_ethylene_). U_iso_(H) values were set to 1.2U_eq_ of the attached carbon atom.

### Crystal data

**Table:**

[Ir(C_9_H_7_)(C_2_H_4_)_2_]	*F*(000) = 1360
*M**_r_* = 363.45	*D*_x_ = 2.286 Mg m^−^^3^
Monoclinic, *P*2_1_/*c*	Mo *K*α radiation, λ = 0.7107 Å
*a* = 7.73182 (11) Å	Cell parameters from 34473 reflections
*b* = 10.77708 (13) Å	θ = 3.1–32.0°
*c* = 25.6818 (5) Å	µ = 12.57 mm^−^^1^
β = 98.4034 (15)°	*T* = 100 K
*V* = 2117.00 (5) Å^3^	Prism, clear orange
*Z* = 8	0.45 × 0.33 × 0.22 mm

### Data collection

**Table:**

Agilent Xcalibur, Sapphire2 diffractometer	6917 independent reflections
Radiation source: Enhance (Mo) X-ray Source	6733 reflections with *I* > 2σ(*I*)
Graphite monochromator	*R*_int_ = 0.027
Detector resolution: 8.3438 pixels mm^-1^	θ_max_ = 32.0°, θ_min_ = 2.9°
ω and π scans	*h* = −11→11
Absorption correction: gaussian (*CrysAlis PRO*; Agilent, 2013)	*k* = −15→15
*T*_min_ = 0.020, *T*_max_ = 0.142	*l* = −38→37
55683 measured reflections	

### Refinement

**Table:**

Refinement on *F*^2^	Primary atom site location: structure-invariant direct methods
Least-squares matrix: full	Secondary atom site location: difference Fourier map
*R*[*F*^2^ > 2σ(*F*^2^)] = 0.024	Hydrogen site location: inferred from neighbouring sites
*wR*(*F*^2^) = 0.055	H-atom parameters constrained
*S* = 1.46	*w* = 1/[σ^2^(*F*_o_^2^) + (0.*P*)^2^ + 12.8207*P*] where *P* = (*F*_o_^2^ + 2*F*_c_^2^)/3
6917 reflections	(Δ/σ)_max_ = 0.001
253 parameters	Δρ_max_ = 1.69 e Å^−^^3^
0 restraints	Δρ_min_ = −2.04 e Å^−^^3^

### Special details

**Table:**

Geometry. All e.s.d.'s (except the e.s.d. in the dihedral angle between two l.s. planes) are estimated using the full covariance matrix. The cell e.s.d.'s are taken into account individually in the estimation of e.s.d.'s in distances, angles and torsion angles; correlations between e.s.d.'s in cell parameters are only used when they are defined by crystal symmetry. An approximate (isotropic) treatment of cell e.s.d.'s is used for estimating e.s.d.'s involving l.s. planes.
Refinement. Refinement of *F*^2^ against ALL reflections. The weighted *R*-factor *wR* and goodness of fit *S* are based on *F*^2^, conventional *R*-factors *R* are based on *F*, with *F* set to zero for negative *F*^2^. The threshold expression of *F*^2^ > σ(*F*^2^) is used only for calculating *R*-factors(gt) *etc*. and is not relevant to the choice of reflections for refinement. *R*-factors based on *F*^2^ are statistically about twice as large as those based on *F*, and *R*- factors based on ALL data will be even larger.

### Fractional atomic coordinates and isotropic or equivalent isotropic displacement parameters (Å^2^)

**Table:**

	*x*	*y*	*z*	*U*_iso_*/*U*_eq_	
Ir1A	0.306000 (16)	0.166573 (12)	0.397616 (5)	0.00973 (3)	
Ir1B	0.187010 (16)	0.186101 (12)	0.088370 (5)	0.01014 (3)	
C1A	0.1325 (5)	0.2945 (3)	0.43319 (14)	0.0147 (6)	
H1A	0.1386	0.3069	0.4693	0.018*	
C1B	0.3647 (5)	0.0581 (4)	0.05469 (15)	0.0177 (7)	
H1B	0.3600	0.0443	0.0187	0.021*	
C2A	0.2281 (5)	0.3620 (3)	0.39842 (15)	0.0155 (7)	
H2A	0.3161	0.4197	0.4085	0.019*	
C2B	0.4684 (5)	0.1505 (4)	0.08498 (15)	0.0165 (7)	
H2B	0.5327	0.2128	0.0716	0.020*	
C3A	0.1632 (5)	0.3244 (3)	0.34461 (15)	0.0146 (6)	
C3B	0.4555 (4)	0.1297 (3)	0.13992 (14)	0.0142 (6)	
C4A	0.2031 (5)	0.3653 (4)	0.29522 (16)	0.0204 (7)	
H4A	0.2846	0.4280	0.2931	0.024*	
C4B	0.5348 (5)	0.1890 (4)	0.18694 (16)	0.0183 (7)	
H4B	0.6135	0.2539	0.1856	0.022*	
C5A	0.1184 (5)	0.3100 (4)	0.25073 (17)	0.0247 (9)	
H5A	0.1417	0.3368	0.2180	0.030*	
C5B	0.4929 (5)	0.1488 (4)	0.23413 (16)	0.0215 (8)	
H5B	0.5450	0.1866	0.2651	0.026*	
C6A	−0.0051 (6)	0.2120 (4)	0.25316 (17)	0.0241 (8)	
H6A	−0.0605	0.1767	0.2221	0.029*	
C6B	0.3727 (6)	0.0515 (4)	0.23698 (16)	0.0235 (8)	
H6B	0.3490	0.0260	0.2698	0.028*	
C7A	−0.0433 (5)	0.1694 (4)	0.30037 (16)	0.0191 (7)	
H7A	−0.1209	0.1038	0.3017	0.023*	
C7B	0.2895 (5)	−0.0069 (4)	0.19267 (17)	0.0200 (7)	
H7B	0.2078	−0.0693	0.1952	0.024*	
C8A	0.0379 (4)	0.2272 (3)	0.34746 (14)	0.0134 (6)	
C8B	0.3318 (4)	0.0306 (3)	0.14296 (14)	0.0136 (6)	
C9A	0.0265 (4)	0.2051 (3)	0.40240 (14)	0.0138 (6)	
H9A	−0.0389	0.1430	0.4155	0.017*	
C9B	0.2694 (5)	−0.0093 (3)	0.08948 (15)	0.0157 (7)	
H9B	0.1828	−0.0681	0.0795	0.019*	
C10A	0.5669 (5)	0.1874 (4)	0.43434 (16)	0.0179 (7)	
H10A	0.5961	0.2653	0.4525	0.022*	
H10B	0.6203	0.1156	0.4531	0.022*	
C10B	−0.0726 (5)	0.1574 (4)	0.05168 (16)	0.0180 (7)	
H10C	−0.1270	0.2252	0.0304	0.022*	
H10D	−0.0994	0.0762	0.0362	0.022*	
C11A	0.5626 (4)	0.1880 (3)	0.37850 (15)	0.0158 (7)	
H11A	0.6129	0.1165	0.3632	0.019*	
H11B	0.5886	0.2663	0.3627	0.019*	
C11B	−0.0704 (4)	0.1672 (3)	0.10706 (15)	0.0156 (6)	
H11C	−0.0953	0.0920	0.1254	0.019*	
H11D	−0.1230	0.2411	0.1196	0.019*	
C12A	0.3065 (5)	−0.0121 (3)	0.43176 (15)	0.0164 (7)	
H12A	0.4091	−0.0343	0.4565	0.020*	
H12B	0.1975	−0.0398	0.4424	0.020*	
C12B	0.1810 (5)	0.3641 (4)	0.05277 (16)	0.0187 (7)	
H12C	0.2885	0.3920	0.0411	0.022*	
H12D	0.0767	0.3846	0.0285	0.022*	
C13A	0.3210 (5)	−0.0231 (3)	0.37742 (16)	0.0163 (7)	
H13A	0.2208	−0.0575	0.3549	0.020*	
H13B	0.4324	−0.0520	0.3689	0.020*	
C13B	0.1702 (5)	0.3777 (3)	0.10754 (15)	0.0155 (6)	
H13C	0.0592	0.4062	0.1166	0.019*	
H13D	0.2710	0.4136	0.1292	0.019*	

### Atomic displacement parameters (Å^2^)

**Table:**

	*U*^11^	*U*^22^	*U*^33^	*U*^12^	*U*^13^	*U*^23^
Ir1A	0.00901 (5)	0.00969 (5)	0.01044 (6)	−0.00009 (4)	0.00123 (4)	0.00033 (4)
Ir1B	0.00871 (5)	0.01052 (5)	0.01127 (6)	0.00065 (4)	0.00171 (4)	0.00062 (4)
C1A	0.0151 (15)	0.0157 (15)	0.0135 (16)	0.0026 (12)	0.0025 (12)	−0.0021 (12)
C1B	0.0170 (16)	0.0220 (18)	0.0145 (17)	0.0068 (13)	0.0036 (13)	−0.0016 (14)
C2A	0.0129 (15)	0.0108 (14)	0.0222 (18)	−0.0001 (12)	0.0009 (13)	−0.0015 (13)
C2B	0.0113 (14)	0.0216 (17)	0.0174 (17)	0.0029 (12)	0.0044 (12)	0.0029 (14)
C3A	0.0132 (14)	0.0140 (15)	0.0166 (17)	0.0050 (12)	0.0016 (12)	0.0007 (13)
C3B	0.0120 (14)	0.0156 (15)	0.0146 (16)	0.0030 (12)	0.0010 (12)	0.0016 (12)
C4A	0.0213 (18)	0.0215 (18)	0.0190 (18)	0.0057 (14)	0.0051 (14)	0.0069 (14)
C4B	0.0142 (15)	0.0195 (17)	0.0197 (18)	0.0022 (13)	−0.0028 (13)	−0.0002 (14)
C5A	0.0223 (19)	0.036 (2)	0.0161 (18)	0.0099 (17)	0.0053 (15)	0.0069 (16)
C5B	0.0211 (18)	0.027 (2)	0.0148 (17)	0.0056 (15)	−0.0036 (14)	−0.0030 (15)
C6A	0.0212 (18)	0.036 (2)	0.0138 (17)	0.0082 (16)	−0.0027 (14)	−0.0037 (16)
C6B	0.026 (2)	0.031 (2)	0.0134 (17)	0.0086 (16)	0.0046 (15)	0.0065 (15)
C7A	0.0136 (15)	0.0221 (18)	0.0201 (18)	0.0024 (13)	−0.0029 (13)	−0.0050 (14)
C7B	0.0170 (16)	0.0198 (17)	0.024 (2)	0.0021 (13)	0.0065 (14)	0.0078 (15)
C8A	0.0106 (14)	0.0145 (15)	0.0146 (16)	0.0018 (11)	0.0008 (12)	−0.0007 (12)
C8B	0.0119 (14)	0.0137 (15)	0.0149 (16)	0.0020 (12)	0.0005 (12)	0.0015 (12)
C9A	0.0115 (14)	0.0157 (15)	0.0146 (16)	0.0010 (12)	0.0029 (12)	0.0002 (12)
C9B	0.0153 (15)	0.0127 (15)	0.0183 (17)	0.0030 (12)	−0.0006 (13)	−0.0013 (13)
C10A	0.0117 (15)	0.0206 (17)	0.0203 (18)	−0.0031 (13)	−0.0017 (13)	0.0045 (14)
C10B	0.0107 (15)	0.0224 (18)	0.0200 (18)	−0.0005 (13)	−0.0004 (13)	0.0029 (14)
C11A	0.0115 (14)	0.0154 (15)	0.0211 (18)	−0.0005 (12)	0.0044 (12)	0.0015 (13)
C11B	0.0103 (14)	0.0158 (15)	0.0211 (18)	−0.0017 (12)	0.0043 (12)	−0.0011 (13)
C12A	0.0181 (16)	0.0116 (15)	0.0190 (18)	−0.0007 (12)	0.0012 (13)	0.0052 (13)
C12B	0.0197 (17)	0.0157 (16)	0.0220 (19)	0.0025 (13)	0.0070 (14)	0.0059 (14)
C13A	0.0167 (16)	0.0107 (14)	0.0220 (19)	−0.0010 (12)	0.0051 (13)	0.0000 (13)
C13B	0.0173 (16)	0.0132 (15)	0.0156 (17)	−0.0004 (12)	0.0014 (13)	−0.0002 (12)

### Geometric parameters (Å, º)

**Table:**

Ir1A—C1A	2.210 (3)	C5A—H5A	0.9300
Ir1A—C2A	2.190 (4)	C5A—C6A	1.430 (7)
Ir1A—C3A	2.349 (4)	C5B—H5B	0.9300
Ir1A—C8A	2.366 (3)	C5B—C6B	1.409 (6)
Ir1A—C9A	2.220 (3)	C6A—H6A	0.9300
Ir1A—C10A	2.109 (4)	C6A—C7A	1.367 (6)
Ir1A—C11A	2.123 (3)	C6B—H6B	0.9300
Ir1A—C12A	2.114 (3)	C6B—C7B	1.374 (6)
Ir1A—C13A	2.115 (4)	C7A—H7A	0.9300
Ir1B—C1B	2.208 (4)	C7A—C8A	1.421 (5)
Ir1B—C2B	2.222 (3)	C7B—H7B	0.9300
Ir1B—C3B	2.371 (3)	C7B—C8B	1.421 (5)
Ir1B—C8B	2.358 (3)	C8A—C9A	1.445 (5)
Ir1B—C9B	2.197 (4)	C8B—C9B	1.451 (5)
Ir1B—C10B	2.111 (4)	C9A—H9A	0.9300
Ir1B—C11B	2.123 (3)	C9B—H9B	0.9300
Ir1B—C12B	2.121 (4)	C10A—H10A	0.9700
Ir1B—C13B	2.129 (4)	C10A—H10B	0.9700
C1A—H1A	0.9300	C10A—C11A	1.428 (5)
C1A—C2A	1.435 (5)	C10B—H10C	0.9700
C1A—C9A	1.426 (5)	C10B—H10D	0.9700
C1B—H1B	0.9300	C10B—C11B	1.422 (5)
C1B—C2B	1.433 (5)	C11A—H11A	0.9700
C1B—C9B	1.434 (5)	C11A—H11B	0.9700
C2A—H2A	0.9300	C11B—H11C	0.9700
C2A—C3A	1.456 (5)	C11B—H11D	0.9700
C2B—H2B	0.9300	C12A—H12A	0.9700
C2B—C3B	1.445 (5)	C12A—H12B	0.9700
C3A—C4A	1.417 (5)	C12A—C13A	1.419 (5)
C3A—C8A	1.435 (5)	C12B—H12C	0.9700
C3B—C4B	1.422 (5)	C12B—H12D	0.9700
C3B—C8B	1.442 (5)	C12B—C13B	1.427 (5)
C4A—H4A	0.9300	C13A—H13A	0.9700
C4A—C5A	1.367 (6)	C13A—H13B	0.9700
C4B—H4B	0.9300	C13B—H13C	0.9700
C4B—C5B	1.368 (6)	C13B—H13D	0.9700
			
C1A—Ir1A—C3A	61.69 (13)	C2B—C3B—Ir1B	66.12 (19)
C1A—Ir1A—C8A	61.10 (13)	C4B—C3B—Ir1B	126.3 (3)
C1A—Ir1A—C9A	37.56 (13)	C4B—C3B—C2B	132.5 (4)
C2A—Ir1A—C1A	38.07 (14)	C4B—C3B—C8B	119.6 (3)
C2A—Ir1A—C3A	37.20 (13)	C8B—C3B—Ir1B	71.75 (19)
C2A—Ir1A—C8A	61.29 (13)	C8B—C3B—C2B	107.9 (3)
C2A—Ir1A—C9A	63.17 (13)	C3A—C4A—H4A	120.9
C3A—Ir1A—C8A	35.43 (12)	C5A—C4A—C3A	118.1 (4)
C9A—Ir1A—C3A	61.18 (13)	C5A—C4A—H4A	120.9
C9A—Ir1A—C8A	36.55 (13)	C3B—C4B—H4B	120.7
C10A—Ir1A—C1A	110.30 (15)	C5B—C4B—C3B	118.7 (4)
C10A—Ir1A—C2A	98.05 (15)	C5B—C4B—H4B	120.7
C10A—Ir1A—C3A	121.30 (14)	C4A—C5A—H5A	119.1
C10A—Ir1A—C8A	156.62 (14)	C4A—C5A—C6A	121.8 (4)
C10A—Ir1A—C9A	146.06 (15)	C6A—C5A—H5A	119.1
C10A—Ir1A—C11A	39.44 (15)	C4B—C5B—H5B	119.2
C10A—Ir1A—C12A	88.13 (15)	C4B—C5B—C6B	121.6 (4)
C10A—Ir1A—C13A	97.41 (15)	C6B—C5B—H5B	119.2
C11A—Ir1A—C1A	132.10 (14)	C5A—C6A—H6A	119.4
C11A—Ir1A—C2A	99.50 (14)	C7A—C6A—C5A	121.2 (4)
C11A—Ir1A—C3A	99.40 (13)	C7A—C6A—H6A	119.4
C11A—Ir1A—C8A	128.14 (14)	C5B—C6B—H6B	119.0
C11A—Ir1A—C9A	160.23 (14)	C7B—C6B—C5B	122.0 (4)
C12A—Ir1A—C1A	111.18 (14)	C7B—C6B—H6B	119.0
C12A—Ir1A—C2A	148.74 (15)	C6A—C7A—H7A	120.6
C12A—Ir1A—C3A	150.56 (14)	C6A—C7A—C8A	118.7 (4)
C12A—Ir1A—C8A	115.17 (14)	C8A—C7A—H7A	120.6
C12A—Ir1A—C9A	95.20 (14)	C6B—C7B—H7B	120.9
C12A—Ir1A—C11A	104.42 (14)	C6B—C7B—C8B	118.1 (4)
C12A—Ir1A—C13A	39.23 (15)	C8B—C7B—H7B	120.9
C13A—Ir1A—C1A	139.61 (14)	C3A—C8A—Ir1A	71.65 (19)
C13A—Ir1A—C2A	162.85 (14)	C3A—C8A—C9A	107.9 (3)
C13A—Ir1A—C3A	126.52 (14)	C7A—C8A—Ir1A	125.8 (3)
C13A—Ir1A—C8A	102.03 (14)	C7A—C8A—C3A	119.6 (3)
C13A—Ir1A—C9A	106.42 (14)	C7A—C8A—C9A	132.5 (3)
C13A—Ir1A—C11A	87.83 (14)	C9A—C8A—Ir1A	66.22 (19)
C1B—Ir1B—C2B	37.74 (14)	C3B—C8B—Ir1B	72.7 (2)
C1B—Ir1B—C3B	61.00 (13)	C3B—C8B—C9B	107.4 (3)
C1B—Ir1B—C8B	61.37 (13)	C7B—C8B—Ir1B	125.7 (3)
C2B—Ir1B—C3B	36.48 (13)	C7B—C8B—C3B	120.0 (3)
C2B—Ir1B—C8B	61.19 (13)	C7B—C8B—C9B	132.5 (3)
C8B—Ir1B—C3B	35.51 (12)	C9B—C8B—Ir1B	65.48 (19)
C9B—Ir1B—C1B	38.00 (14)	Ir1A—C9A—H9A	118.0
C9B—Ir1B—C2B	63.37 (14)	C1A—C9A—Ir1A	70.85 (19)
C9B—Ir1B—C3B	61.27 (13)	C1A—C9A—C8A	108.5 (3)
C9B—Ir1B—C8B	36.94 (13)	C1A—C9A—H9A	125.7
C10B—Ir1B—C1B	109.80 (15)	C8A—C9A—Ir1A	77.2 (2)
C10B—Ir1B—C2B	146.00 (15)	C8A—C9A—H9A	125.7
C10B—Ir1B—C3B	155.32 (14)	Ir1B—C9B—H9B	117.0
C10B—Ir1B—C8B	119.92 (14)	C1B—C9B—Ir1B	71.4 (2)
C10B—Ir1B—C9B	96.90 (15)	C1B—C9B—C8B	108.0 (3)
C10B—Ir1B—C11B	39.26 (15)	C1B—C9B—H9B	126.0
C10B—Ir1B—C12B	88.89 (15)	C8B—C9B—Ir1B	77.6 (2)
C10B—Ir1B—C13B	99.14 (15)	C8B—C9B—H9B	126.0
C11B—Ir1B—C1B	133.24 (15)	Ir1A—C10A—H10A	116.5
C11B—Ir1B—C2B	161.22 (14)	Ir1A—C10A—H10B	116.5
C11B—Ir1B—C3B	128.47 (14)	H10A—C10A—H10B	113.5
C11B—Ir1B—C8B	100.21 (13)	C11A—C10A—Ir1A	70.8 (2)
C11B—Ir1B—C9B	100.64 (14)	C11A—C10A—H10A	116.5
C11B—Ir1B—C13B	87.13 (14)	C11A—C10A—H10B	116.5
C12B—Ir1B—C1B	111.80 (15)	Ir1B—C10B—H10C	116.5
C12B—Ir1B—C2B	95.70 (14)	Ir1B—C10B—H10D	116.5
C12B—Ir1B—C3B	115.72 (14)	H10C—C10B—H10D	113.5
C12B—Ir1B—C8B	151.18 (14)	C11B—C10B—Ir1B	70.8 (2)
C12B—Ir1B—C9B	149.22 (15)	C11B—C10B—H10C	116.5
C12B—Ir1B—C11B	102.79 (15)	C11B—C10B—H10D	116.5
C12B—Ir1B—C13B	39.24 (15)	Ir1A—C11A—H11A	116.7
C13B—Ir1B—C1B	139.24 (15)	Ir1A—C11A—H11B	116.7
C13B—Ir1B—C2B	105.64 (14)	C10A—C11A—Ir1A	69.7 (2)
C13B—Ir1B—C3B	101.37 (13)	C10A—C11A—H11A	116.7
C13B—Ir1B—C8B	126.20 (13)	C10A—C11A—H11B	116.7
C13B—Ir1B—C9B	162.21 (14)	H11A—C11A—H11B	113.7
Ir1A—C1A—H1A	123.7	Ir1B—C11B—H11C	116.6
C2A—C1A—Ir1A	70.2 (2)	Ir1B—C11B—H11D	116.6
C2A—C1A—H1A	126.2	C10B—C11B—Ir1B	69.9 (2)
C9A—C1A—Ir1A	71.6 (2)	C10B—C11B—H11C	116.6
C9A—C1A—H1A	126.2	C10B—C11B—H11D	116.6
C9A—C1A—C2A	107.7 (3)	H11C—C11B—H11D	113.6
Ir1B—C1B—H1B	123.5	Ir1A—C12A—H12A	116.6
C2B—C1B—Ir1B	71.6 (2)	Ir1A—C12A—H12B	116.6
C2B—C1B—H1B	125.9	H12A—C12A—H12B	113.6
C2B—C1B—C9B	108.1 (3)	C13A—C12A—Ir1A	70.4 (2)
C9B—C1B—Ir1B	70.6 (2)	C13A—C12A—H12A	116.6
C9B—C1B—H1B	125.9	C13A—C12A—H12B	116.6
Ir1A—C2A—H2A	117.0	Ir1B—C12B—H12C	116.5
C1A—C2A—Ir1A	71.7 (2)	Ir1B—C12B—H12D	116.5
C1A—C2A—H2A	125.9	H12C—C12B—H12D	113.5
C1A—C2A—C3A	108.1 (3)	C13B—C12B—Ir1B	70.7 (2)
C3A—C2A—Ir1A	77.4 (2)	C13B—C12B—H12C	116.5
C3A—C2A—H2A	125.9	C13B—C12B—H12D	116.5
Ir1B—C2B—H2B	117.9	Ir1A—C13A—H13A	116.6
C1B—C2B—Ir1B	70.6 (2)	Ir1A—C13A—H13B	116.6
C1B—C2B—H2B	126.0	C12A—C13A—Ir1A	70.4 (2)
C1B—C2B—C3B	108.0 (3)	C12A—C13A—H13A	116.6
C3B—C2B—Ir1B	77.4 (2)	C12A—C13A—H13B	116.6
C3B—C2B—H2B	126.0	H13A—C13A—H13B	113.6
C2A—C3A—Ir1A	65.45 (19)	Ir1B—C13B—H13C	116.6
C4A—C3A—Ir1A	126.7 (3)	Ir1B—C13B—H13D	116.6
C4A—C3A—C2A	132.2 (4)	C12B—C13B—Ir1B	70.1 (2)
C4A—C3A—C8A	120.5 (3)	C12B—C13B—H13C	116.6
C8A—C3A—Ir1A	72.9 (2)	C12B—C13B—H13D	116.6
C8A—C3A—C2A	107.2 (3)	H13C—C13B—H13D	113.6

### Selected Ir to indenyl bond distances Å.

**Table:**

Ir1A—C1A	2.210 (3)	Ir1B—C1B	2.208 (4)
Ir1A—C2A	2.190 (4)	Ir1B—C2B	2.222 (3)
Ir1A—C3A	2.349 (4)	Ir1B—C3B	2.371 (3)
Ir1A—C8A	2.366 (3)	Ir1B—C8B	2.358 (3)
Ir1A—C9A	2.220 (3)	Ir1B—C9B	2.197 (4)
